# Effect of Weather on Frequency of Vaso-Occlusive Crisis in Children With Sickle Cell Disease

**DOI:** 10.7759/cureus.17254

**Published:** 2021-08-17

**Authors:** Mohamed Almuqamam, Kanya Ahuja, Inas Wassef, Sasikumar Kilaikode, Aziza Sedrak

**Affiliations:** 1 Pediatrics, The Brooklyn Hospital Center, Brooklyn, USA

**Keywords:** humidity, sickle cell disease, temperature, vaso-occlusive crisis, weather

## Abstract

Introduction

Sickle cell disease (SCD) is characterized by acute vaso-occlusive crisis (VOC) often manifested as painful episodes. Environmental factors are known to play a role in the frequency and severity of VOC.

Methods

The aim of this study is to analyze the relationship between weather changes and VOC in children with SCD. Data on daily temperature, humidity, and wind speed in Brooklyn, New York was collected over one year. Daily census data of children < 20 years of age with SCD presenting with VOC during the study period was retrieved from the Health Information Systems database. Data was analyzed to determine correlations of daily temperature, humidity, and wind speed with the number of VOCs using Pearson correlation co-efficient and time-series statistics.

Results

The total number of episodes of VOC was 344, with 218 outpatients and 126 inpatients. Total episodes of VOC peaked during January (n=44), while they were lowest in July (n=16). We observed a negative correlation of VOC with temperature (r= -0.05, p=0.04) and no correlation with humidity (r=0.01, p=0.85) was noted. Analysis of wind speed showed a negative correlation with VOC which is not significant.

Conclusion

No significant correlation was found between changes in humidity or wind speed and VOC. As this study was performed in an urban environment with extreme weather changes, results may be different in other geographic areas.

## Introduction

Sickle cell disease (SCD) is a common inherited disorder, characterized by hemolytic anemia, vaso-occlusive episodes, and acute and chronic organ damage leading to shortened life span. The most characteristic symptom is acute pain crisis which accounts for significant morbidity in patients with SCD. Acute pain is caused primarily by vaso-occlusion, which is a complex process involving red cells, white blood cells, platelets, and vascular endothelium with nitric oxide playing a central role [1]. The frequency of painful episodes varies between patients. The reason for this variability is unknown with a possible role of environmental and genetic factors.

Studies were done previously to clarify the effect of weather changes on the frequency and severity of pain crises in patients with SCD [2-5]. Various studies demonstrated positive [4,5] and negative [2] correlations, the majority of which were done in adult patients. It is, therefore relevant to study the possible effect of weather on the occurrence of acute vaso-occlusive crisis (VOC) among children with SCD.

## Materials and methods

This is a prospective study done from July 26 2011 to July 31 2012 at a university-affiliated community hospital. Appropriate approval was obtained from the Institutional Review Board. VOC was described as painful episodes without associated complications like infections or acute chest syndrome. Patients with SCD ranging in age from two to twenty years of age with a pain score of six out of ten or more or requiring intravenous analgesics treated in pediatric hematology/oncology facility or admitted to the inpatient service were included in our study. Pain was scored using the universal pain assessment tool. Patients who received oral analgesics or with a pain score of less than six out of ten were excluded.

Demographic characteristics (age, sex, and ethnicity) and clinical data were retrieved from our Health Information Systems database. Weather changes including temperature, humidity, and wind speed (average for that day) were collected from the Weather Channel website. Data were analyzed to determine correlations of daily temperature, humidity, and wind speed with VOC using Pearson correlation co-efficient and time-series statistics. Statistical Package for the Social Sciences (SPSS) software version 25.0 (IBM Corp., Armonk, NY) was used for the analysis. P-value < 0.05 was considered signiﬁcant.

## Results

Our study included a total of 70 patients with 344 episodes of VOC. Out of 344 episodes, 184 (53.5%) were males and 160, (46.5%) females. Race distribution of the episodes was as follows: 297 (86.3%) African Americans, 28 (8.1%) Hispanics, and 19 (5.6%) others. The age of the patients varied from two to twenty years. The demographic profile is shown in Table 1.

**Table 1:** Demographic variables

Age	2-7y	58(16.9%)
	8-12y	60 (17.5%)
	13-17 y	184 (53.4%)
	18-20y	42 (12.2%)
Gender	male	184 (53.5%)
	Female	160 (46.5%)
Ethnicity	African American	297(86.3%)
	Hispanic	28(8.1%)
	Other	19(5.6%)

Out of the 344 episodes, 218 (63.4%) were treated in the pediatric hematology/oncology outpatient facility and 126 (36.6%), in the inpatient unit. The average duration of treatment as an outpatient was six hours. The peak incidence of VOC occurred during the month of January (12.8%), while they were lowest in July (4.65%). The highest mean temperature was noticed in the month of July 2012 (87.5±6.7 0F) and the lowest in the month of January 2012 (37.9±10.4 0F).

**Figure 1:**
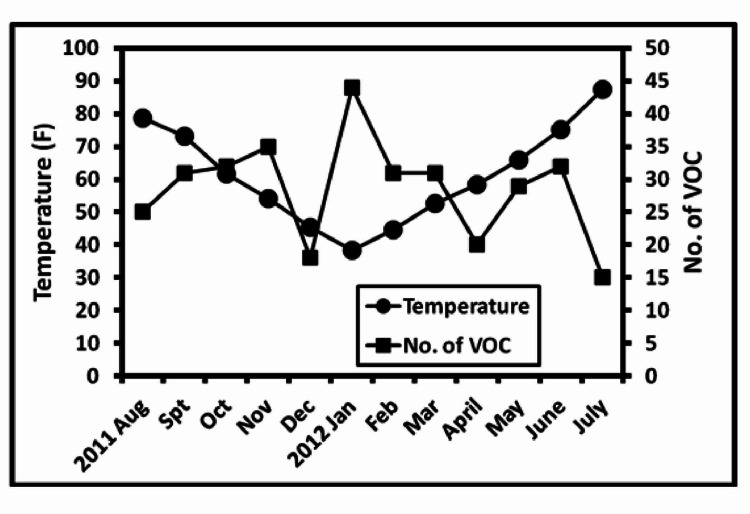
Average monthly temperature compared with the number of vaso-occlusive crisis (VOC) in each month

We observed a negative correlation of VOC with temperature (r= -0.05, p=0.04). No correlation with humidity (r=0.01, p=0.85) was noted. Analysis of the relation of wind speed to the number of VOC showed a negative correlation which was not statistically significant (r= -0.5, p=0.61). The results are shown in Table 2 and Figure 1. 

**Table 2:** Average monthly weather parameters compared to the number of VOC VOC: vaso-occlusive crisis. Mph: miles per hour

Month	Temperature ( in ^0^F)	Humidity (%)	Wind speed(mph)	Number of VOC
2011 Aug	78.74	61.8	4.53	25
Sept	73.2	69.4	4.46	31
Oct	61.6	55.3	5.38	32
Nov	54.2	58.1	5.7	35
Dec	45.3	55.5	5.87	18
2012 Jan	38.45	55.3	7.79	44
Feb	44.5	52.5	6.68	31
Mar	52.58	56	5.77	31
April	58.4	42.5	6	20
May	65.9	63.8	3.96	29
June	75.1	57.4	4.46	32
July	87.5	53.7	4.18	15

## Discussion

Patients with SCD experienced VOC throughout the year, but more in the winter months. The peak incidence was in January, which coincided with the lowest average temperatures of the year. The association between cold weather and VOC has been demonstrated by previous authors [3-5]. The mechanism underlying the pathogenesis of cold-induced VOC in SCD is probably related to abnormal neurovascular reflexes leading to intra-medullary vasoconstriction and marrow infarcts. It was shown by in vitro studies that lowering of the temperature leads to an increased viscosity of sickle-cell blood, which could contribute to stasis of the circulation in peripheral capillary beds [6]. Brandow et al. observed that SCD patients have increased pain sensitivity to both cold and heat compared to healthy race-matched controls [7].

Studies done previously looking at the effect of humidity on VOC showed varying results. We observed no significant effect of humidity on VOC which is consistent with observations by Slovis et al. [2]. In contrast, a study done at King’s College Hospital, London found that windy weather and low humidity were associated with an increased number of episodes of VOC but showed no relationship to temperature, rainfall, and barometric pressure [8]. We found there was no significant relationship between the number of VOC and the wind speed. Nolan et al. showed that there is a positive association between wind speed and the number of painful episodes in SCD patients. This may be due to the fact that skin cooling is associated with sickle vaso-occlusion and perhaps, pain [9].

A review of the results from published studies looking at the association of weather changes and VOC has been inconsistent. The range of cold temperatures in the studies which had positive [4-5] and negative [2,8] correlations did not differ significantly from our own findings. Similarly, the changes in wind speed and humidity also showed discordant results. These findings suggest that though the lower temperatures may play a role in inducing VOC in patients with SCD, as yet undetermined additional factors are involved in the development of VOC symptoms.

## Conclusions

In summary, we observed that the number of VOC increased in colder months with a peak in January among children with SCD. No significant correlation was found between changes in humidity or wind speed and VOC. As this study was performed in an urban environment with extreme weather changes, results may be different in other geographic areas. Further studies involving a larger cohort are needed to clarify the effect of weather on VOC. Additional confounding factors may play a role in the initiation of VOC in SCD.
